# Primary bronchial myeloid sarcoma mimicking bronchogenic carcinoma: a case report

**DOI:** 10.1186/s12890-019-0975-7

**Published:** 2019-11-08

**Authors:** Wang Wang, Hesheng He, Xingwu Chen, Chenhong Zhang

**Affiliations:** 1 Department of Internal Medicine, the First Affiliated Hospital of Wannan Medical College, Zheshan West Road, Wuhu, 241000 China; 2 Department of Hematology, the First Affiliated Hospital of Wannan Medical College, Zheshan West Road, Wuhu, 241000 China; 3 Department of Respiration, the First Affiliated Hospital of Wannan Medical College, Zheshan West Road, Wuhu, 241000 China

**Keywords:** Bronchus, Carcinoma, Pulmonary mass, Myeloid sarcoma

## Abstract

**Background:**

Myeloid sarcoma (MS) rarely involves the bronchus, and primary bronchial MS has almost never been reported in mainland China.

**Case presentation:**

A 65-year-old female patient was admitted with a 3-month history of cough. She was initially diagnosed with bronchogenic carcinoma according to chest computed tomography (CT). However, after a biopsy was taken from the endobronchial lesion by bronchoscopy and further immunohistochemical analysis was performed, the diagnosis of MS was made. Because her bone marrow was normal and she had no history of haematologic diseases, we further considered the diagnosis of primary bronchial MS. The patient received chemotherapy with HAG regimens, and the original mass completely resolved, as confirmed by chest CT scan after 3 cycles of treatment. Meanwhile, no abnormalities were found on re-examination via bronchoscopy.

**Conclusions:**

MS should be considered in the differential diagnosis in the presence of a suspicious pulmonary mass. Immunohistochemical analysis is necessary to confirm the diagnosis. Chemotherapy can lengthen the survival time for patients.

## Background

Myeloid sarcoma (MS), a solid mass composed of immature myeloid cells, is uncommon in clinical practice. Although it can occur in some patients with acute myeloid leukaemia (AML), there are also a few cases without bone marrow involvement, presenting only as a locally isolated mass, called primary MS [1]. Among MS patients, bronchus involvement is rather uncommon; here, we report a rare case of primary bronchial MS with cough as the first symptom.

## Case presentation

A 65-year-old female, who was previously healthy, was admitted to the respiratory department of our hospital with a 3-month history of cough. She complained of repeatedly coughing without any inducement, accompanied by a small amount of white foamy phlegm. A dull pain in the left lower chest may have occurred when the cough was severe. Although the chest radiograph performed at the local hospital was normal, she received antibiotic treatment for 8 days. Unfortunately, her symptoms of cough and phlegm were aggravated after treatment.

**Fig. 1:**
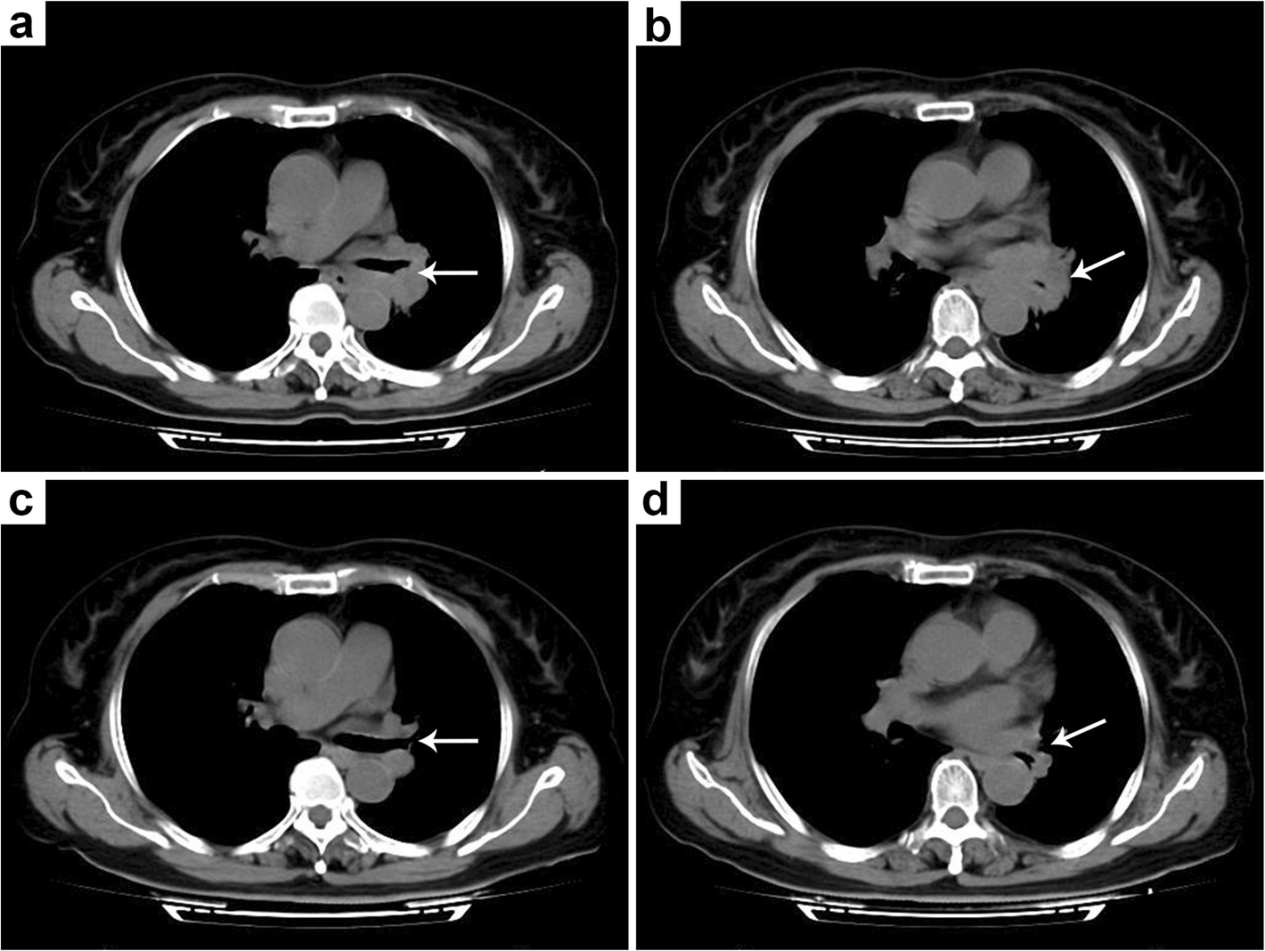
Four computed tomography (CT) images of the chest before and after treatment. **a** The left upper and lower lobar bronchi at the entrance are obstructed (arrow). **b** A mass in the left lung hilar region (arrow), which is indistinguishable from the surrounding tissue. **c** The original obstructed bronchi are unobstructed after treatment (arrow). **d** The original mass disappeared after treatment (arrow), and the thoracic aorta and pulmonary vessels are clearly defined

**Fig. 2:**
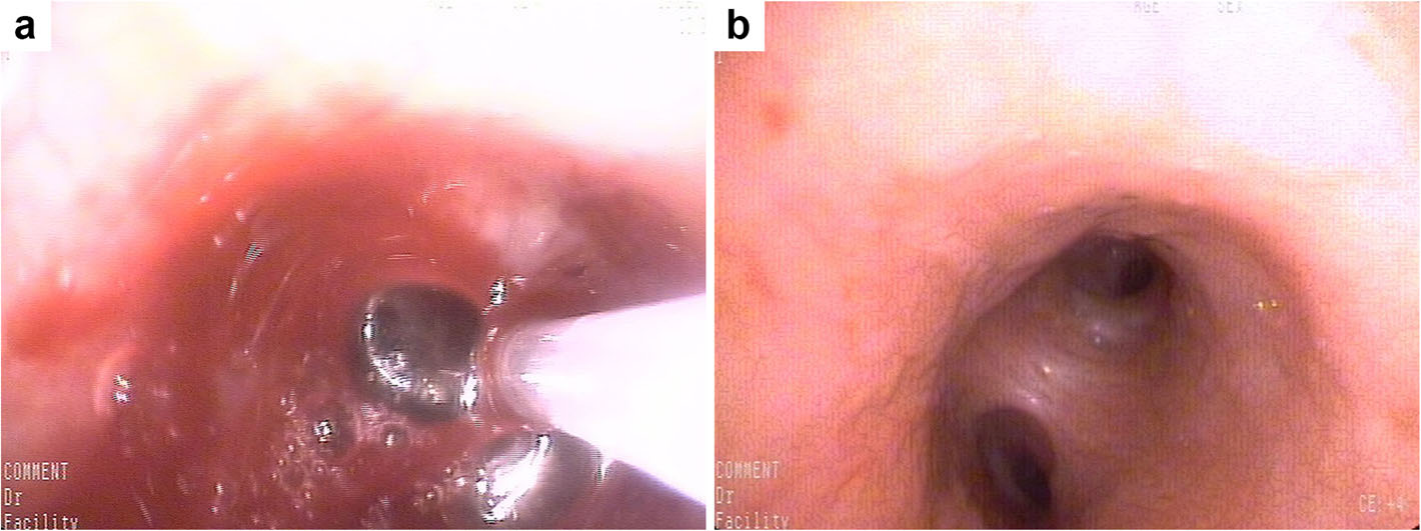
Bronchoscopic pictures before and after the treatment. **a** The patient underwent bronchoscopy showing endobronchial neoplasm obstructing the left main bronchus, with a biopsy taken from the lesion by forceps. **b** No abnormalities were found on re-examination via bronchoscopy after treatment

**Fig. 3:**
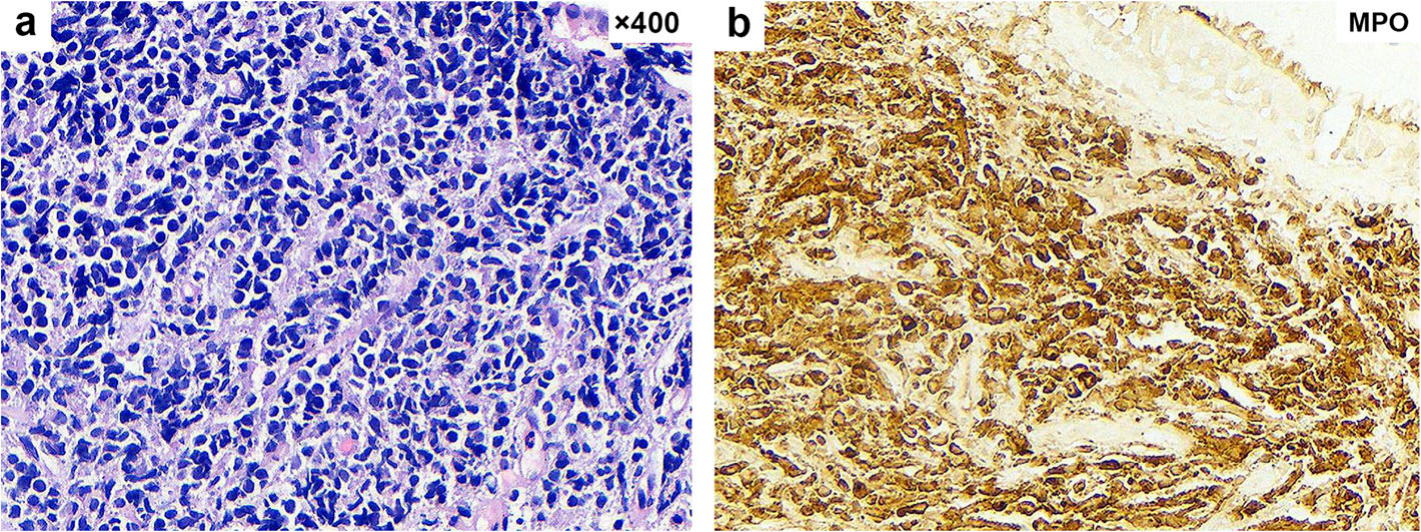
Pathological findings of the endobronchial biopsy. **a** The cytoplasm of the tumour cells is partially basophilic with large round, oval or lobulated nuclei in which 1–2 small nucleoli can be found (haematoxylin and eosin, × 400). **b** Immunochemistry showed positive staining for myeloperoxidase (MPO) in neoplastic cells

The chest computed tomography (CT) scan of the patient revealed a left hilar mass (Fig. 1a, b); she subsequently underwent bronchoscopy showing endobronchial neoplasm obstructing the left main bronchus (Fig. 2a), with a biopsy taken from the lesion. The biopsy pathology showed that the bronchial submucosa was uniformly diffuse with small- to medium-sized lymphoid cells at low magnification (× 100). At high magnification (× 400), the cytoplasm of the tumour cells was observed to be partially basophilic with large round, oval or lobulated nuclei in which 1–2 small nucleoli could be found (Fig. 3a). Based on these findings, haematological diseases were considered. The patient was transferred to the haematology department for further diagnosis and treatment.

During the physical examination in the haematology department, there was no evidence of fever, pale appearance, subcutaneous bleeding, hepatosplenomegaly or superficial lymph node enlargement. Sternal tenderness was negative. Respiratory sounds in the left lung were weakened, and no dry or wet rales were detected in either side of the lung. No abdominal mass was detected. Neurological examination showed no abnormalities.

The complete blood count was normal. Bone marrow biopsy and chromosome analysis did not show any additional abnormalities. Immunochemistry revealed the expression of MPO, CD68, CD15, CD117, CD34, lysozyme, CD99, CD43, CD56, and Ki-67 (70%) (Fig. 3b). In addition, expression was negative for CD3, CD5, CD20, and CD79a. Synthesizing the characteristics on chest CT and the results of the bronchoscopy and immunohistochemical analysis, we made the diagnosis of MS, which was further confirmed to be primary bronchial MS based on the normal bone marrow and lack of a history of haematological disorders.

After assessing the patient’s condition and obtaining her consent, chemotherapy with HAG (Homoharringtonine, Cytarabine, G-CSF) regimens was begun. Upon the completion of 3 cycles of treatment, the original mass had completely resolved, as confirmed by chest CT scan (Fig. 1c, d). No abnormalities were found on re-examination via bronchoscopy (Fig. 2b). However, we still obtained some local endobronchial mucosa from the original lesion, and the pathological results were normal. Routine blood and bone marrow examinations were performed again, which showed no abnormalities. No positive signs were found on the physical examination. The patient was discharged from our department without any symptoms.

One year later, the patient was re-examined in the local hospital and no abnormalities were found on routine blood tests, bone marrow biopsy, chest CT or bronchoscopy.

### Immunohistochemistry data

MPO(polyclone), CD68(KP1), CD15(Carb-3), CD117(2E4),

CD34(QBEnd/10), Lysozyme(polyclone), CD99(O13),

CD43(DF-T1), CD56(123C3.D5), Ki-67(70%)(MX006).

The immunohistochemical analysis was performed on Ventana BenchMark GX System.

All materials used for the test were purchased from Fuzhou Maixin Biotechnology Development Co.,LTD.

## Discussion

MS, also called “chloroma” or “granulocytic sarcoma”, is an extramedullary mass composed of medullary primitive cells. The first reported case of MS dates back to 1811 and was described by Burns, who states that the cut surface of the mass after surgery appeared green. Subsequent studies showed that it is closely related to myeloperoxidase from tumour cells. MS has a close relationship with AML. It can occur before, after or concurrent with AML. The incidence of MS is very low (2/100,000 in adults and 0.7/100,000 in children) [2]. The most common sites of involvement are mainly the skin, lymph nodes and bones, while bronchus involvement is rare. Therefore, the diagnosis of this disease is challenging.

At present, there is a lack of prospective research on this study. Retrospective analysis is also rare, and most studies have been case reports. MS is often difficult to detect early, unless there is a clinical manifestation of mass invasion in a particular part of the body. In this case, the patient was initially admitted for recurrent cough, which was later found to be caused by primary bronchial MS that was finally confirmed through comprehensive analysis of chest CT, bronchoscopy, bone marrow examination and immunohistochemistry results.

The diagnosis of MS is very difficult, and the initial misdiagnosis rate is high. Neiman [3] clearly noted in his analysis of 61 cases of MS that the initial diagnosis of MS was only approximately 44% accurate. Based on clinical experience and existing research, the diagnosis of MS requires comprehensive analyses, including clinical manifestations, imaging examinations, laboratory examinations and immunohistochemistry; immunohistochemistry is the most important part of the final diagnosis. With the development of diagnostic immunohistochemical techniques, the diagnosis rate of this disease has been greatly improved. Because MPO, CD43, CD68, CDl5, CD117, lysozyme and so on can be expressed by myeloid cells, they are considered reliable markers for the identification of MS, and the detection of their positive expression provides great assistance in the diagnosis of the disease. MPO, in particular, is a specific antigen marker of MS, which is very important for the diagnosis and differential diagnosis of MS. The negative B cell markers (CD20) also favor the diagnosis of MS [4]. For the diagnosis of primary MS, it is generally thought that a patient can be diagnosed if the bone marrow is not involved and the patient lacks a history of haematological disorders. In this case, the patient was pathologically diagnosed with bronchial MS after admission, with normal bone marrow and no previous haematologic diseases; therefore, the diagnosis of primary bronchial MS was clear.

The treatment of MS includes combination chemotherapy, local surgical resection, radiotherapy and haematopoietic stem cell transplantation. According to existing cases, most patients with MS inevitably develop AML within a few months or years. Therefore, it is recommended MS should be treated in accordance with the treatment of AML [5], with a preference for anthracycline combined with cytarabine chemotherapy regimens. Although there is no evidence that this can effectively prevent MS from converting to AML, it can effectively prolong asymptomatic survival time for patients with MS. Regarding surgery and radiotherapy, some studies have noted that surgery and radiotherapy can only temporarily alleviate the condition of patients but cannot significantly improve the prognosis [6]. The curative effect of haematopoietic stem cell transplantation in the treatment of MS is gradually being recognized by the public, and related studies have shown that the 5-year overall survival rate and leukaemia-free survival rate of MS patients who received haematopoietic stem cell transplantation were 48 and 36%, respectively [7]. Therefore, haematopoietic stem cell transplantation may be a very effective means of treating MS and improving the prognosis.

## Conclusions

MS rarely involves the bronchus. It is very easy to misdiagnose pulmonary cancer based only on clinical manifestations, chest imaging or bronchoscopy. The final diagnosis of MS depends on immunohistochemistry. An early definitive diagnosis and timely and effective chemotherapy can improve the long-term survival rate, and haematopoietic stem cell transplantation should be carried out in the disease remission period if conditions permit to obtain a longer survival period.

## Data Availability

All data supporting our findings is contained within the manuscript.

## Abbreviations

AML: Acute myeloid leukemia
CT: Computed tomography
G-CSF: Granulocyte colony-stimulating factor
HAG: Homoharringtonine, cytarabine
MPO: Myeloperoxidase
MS: Myeloid sarcoma
